# The Accuracy and Reliability of the Power Measurements of the TACX Neo 2T Smart Trainer and Its Agreement against the Garmin Vector 3 Pedals

**DOI:** 10.3390/jfmk9030138

**Published:** 2024-08-17

**Authors:** Jorge E. Morais, José A. Bragada, Pedro M. Magalhães, Daniel A. Marinho

**Affiliations:** 1 Department of Sports Sciences, Instituto Politécnico de Bragança, 5300-253 Bragança, Portugal; jbragada@ipb.pt (J.A.B.); pmaga@ipb.pt (P.M.M.); 2 Research Centre for Active Living and Wellbeing (LiveWell), Instituto Politécnico de Bragança, 5300-253 Bragança, Portugal; 3 Department of Sports Sciences, University of Beira Interior, 6201-001 Covilhã, Portugal; marinho.d@gmail.com; 4 Research Centre in Sports, Health and Human Development (CIDESD), 4960-320 Covilhã, Portugal

**Keywords:** cycling power, power gear, power training, power measurement, pedal power, metrological characteristics

## Abstract

The power output in cycling is one of the most important factors for athletes and coaches. The cycling community has several commercial gears that can be used. One of the most used is the TACX Neo 2T (TN2T) smart trainer. The objective of this study was to investigate the metrological proprieties of the TN2T (accuracy and reliability), as well as its agreement with the Garmin Vector 3 (GV3) pedals at different power stages. The sample consisted of ten regional-level cyclists with a mean age of 45.6 ± 6.4 years, who regularly participated in regional and national competitions. Residual relative differences were found between the two devices. Both devices showed good reliability with coefficients of variation and intraclass correlation coefficients ranging from 0.03% to 0.15% and from 0.731 to 0.968, respectively. Independent samples *t*-test comparison between devices showed no significant differences in all power stages (*p* > 0.05). Bland–Altman plots showed that more than 80% of the plots were within the 95% confidence intervals in all power stages. The present data showed that there were non-significant differences between the two devices at power stages between 100 W and 270 W, with a strong agreement. Therefore, they can be used simultaneously.

## 1. Introduction

Cycling, like any other time-based sport, is strongly dependent on speed where athletes aim to cover a given distance as fast as possible. From a cycling performance perspective, the power output is one of the most important factors for athletes and coaches [1,2]. This provides an objective and quantifiable measure of a cyclist’s performance. Unlike speed, the power output is not influenced by external factors such as wind or terrain [3]. Consequently, monitoring the power output allows cyclists to maintain consistent effort levels, leading to more effective and efficient workouts. Additionally, by tracking the power output over time, cyclists and coaches can monitor the progress, make data-driven adjustments to training plans, and set realistic goals [3]. The power output can be measured during field tests [4,5] or in laboratory settings [6,7].

Irrespective of being in field or laboratory settings, the power output is measured using power meters. These are devices that quantify the power a cyclist generates while riding [8]. Therefore, power meters are widely used in cycling. These devices allow coaches to create and monitor individualized training plans based on power output obtained during training and competition [8]. Due to the meaningful interest in power measurements, there is a vast list of systems available for this purpose. The main methods and devices to measure the power output are (i) crank-based power meters, which are devices integrated into the crank arms or the chainring spider (e.g., SRM crankset or Stages) [7]; (ii) pedal-based devices, which are integrated into the pedals and measure the force applied to the pedals (e.g., and Garmin Vector 3–GV3 or Powertap P1) [9,10]; (iii) hub-based devices, which are located in the rear hub of the wheel and measure the torque applied to the rear wheel (e.g., Powertap G3) [11]; (iv) chainring-based power meters, which are integrated into the chainrings or spider and measure the force applied to the chainrings (e.g., Power2Max or ROTOR INpower) [11,12]; (v) bottom bracket-based devices, which are integrated into the bottom bracket and measure the force applied to the crank arms (e.g., Shimano Dura-Ace, in older models) [4]; and (vi) smart trainers, which are indoor trainers that can measure power output while riding in a stationary way (e.g., TACX Neo 2T–TN2T or Elite Direto) [13,14]. Additionally, there is also the Lode Excalibur device, which is a type of stationary cycling ergometer. This is considered the gold standard device for measuring the power output [15]. In summary, there are laboratory-based ergometers, smart trainers, and mobile power devices with which athletes can measure and control the power output. The latter devices allow coaches and athletes to monitor the latter training load in any training environment. Irrespective of the type of device, the validity of these kinds of power meters has been tested scientifically to provide deeper insights regarding their metrological properties [16]. Commonly, such metrological properties that researchers analyze are related to accuracy, sensitivity, repeatability, reproducibility, and robustness [16].

The TN2T is a smart trainer that is often used by teams and cyclists to monitor training loads. The brand acknowledges that to optimize power efficiency, from the pedal to the motor, there are no physical transmissions (such as a belt, roller, or wheel). Thus, the brand claims that (i) there is no loss of power because there are no disruptions or imperfections that interfere with the cyclist’s power flow, (ii) it does not require calibration, and (iii) it measures performance with an accuracy of 1% (this information was retrieved from the official brand website (https://www.garmin.com/en-US/garmin-technology/tacx/neo-principles/) (accessed 6 August 2024). Additionally, this smart trainer derives its strength from an electromotor containing 32 precisely positioned magnets that rotate around 30 coils; the higher the current through the coils, the larger the magnetic force, and the higher the brake power. Its motor can generate a maximum power of 2500 W with a torque of 85 N/m, enabling high resistance at low speeds (https://www.garmin.com/en-US/garmin-technology/tacx/neo-principles/) (accessed 6 August 2024). As with any smart trainer, it depends on an application to input the training intensity. Therefore, evidence is needed to understand its accuracy and reliability with the intensity preset (i.e., target) in the mobile application (APP), and its agreement against other devices that have already been proven to be accurate and reliable ergometers in submaximal tests, such as the GV3 pedals [9]. As far as our understanding goes, and as indicated by others [17], there is no information about this topic regarding this smart trainer. Therefore, the objective of this study was to investigate the metrological proprieties such as accuracy and reliability, as well as its agreement with the GV3 pedals, thus providing relevant information on the utilization of this device to other researchers.

## 2. Materials and Methods

### 2.1. Participants

The participants consisted of ten regional-level male cyclists with a mean age of 45.6 ± 6.4 years, body mass of 74.7 ± 8.4 kg, height of 173.3 ± 5.1 cm, and 12.4 ± 9.1 years of regular competition. Their maximum oxygen uptake at the time of data collection was 61.9 ± 3.4 mL/kg/min with a power of 237.3 ± 28.4 W at a blood lactate concentration of 4 mmol/L. They regularly competed in regional and national road and mountain bike competitions (Tier 2 athletes) [18]. The study’s inclusion criteria were as follows: (i) participants needed to be over 18 years of age, (ii) individuals participating in at least two cycling training sessions per week, and (iii) they had to be fully fit (without health limitations) and not taking any regular medication [19]. All procedures followed the Declaration of Helsinki regarding human research, and the participants signed an informed consent form. The research design (No 137/2023) was approved by the Polytechnic Ethics Board.

### 2.2. Data Collection

All tests were conducted using the athletes’ own bicycles, which were placed on a TN2T cycling smart trainer (Garmin, Olathe, KS, USA). The bikes were also equipped with GV3 power pedals (Garmin, Olathe, KS, USA) installed and zeroed according to the manufacturer’s guidelines. Both devices were synchronized with each other in time, i.e., each device was linked to its mobile application, and the start was at the same time. Data were downloaded from the Garmin Connect software (v. 5.4.0.23, which stores data from both devices) to a personal computer and afterward aligned within a Microsoft Excel spreadsheet (Microsoft, Microsoft 365, Washington, DC, USA). The participants wore their regular textile jerseys and bib shorts, as well as their cycling shoes. These were equipped with SPD Shimano cleats (SM-SH11, WP-Y42U98010, Japan). Conditions were maintained with an ambient temperature of 20–22 °C and relative humidity of 45–55% throughout data collection. Participants were instructed not to eat for two hours before the tests and to drink only water for hydration purposes. After a 10 min warm-up at a self-selected cadence, the participants underwent an incremental power stage test. This study was part of another laboratory study designed to measure the participants’ power at a blood lactate concentration (measured with a Lactate Pro 2 analyzer–Arkray Inc., Kyoto, Japan) of 4 mmol/L for training purposes [19]. They started the incremental test at a power stage of 100 W. Power was increased every three minutes until the participants exceeded a blood lactate concentration of 4 mmol/L (which was achieved at 270 W). Thus, the power measurements ranged between 100 and 270 W (only nine athletes reached the last stage, i.e., 270 W). Cadence was also monitored and recorded by the system. At each power stage, data for analysis were retrieved from an intermediate minute. This was done to obtain the most stable data possible due to the power stage transitions. Afterward, the average was used for analysis.

### 2.3. Statistical Analysis

The mean plus one standard deviation (SD) was calculated as descriptive statistics. The coefficient of variation (CV) was used to calculate the power variation within each power stage. This was calculated as follows: CV = one standard deviation/mean 100 (%). The accuracy of the smart trainer about the target power preset in the APP and between the two devices was calculated based on the relative difference (in %). Reliability between devices was measured with the CV and the intraclass correlation coefficient (ICC). For the CV, this was deemed as follows: (i) excellent if CV ≤ 10%; (ii) good if CV between 10 and 20%; (iii) acceptable if CV between 20 and 30%; and (iv) poor if CV >30% [20]. For the ICC, the two-way mixed model with an “absolute agreement” definition was used [21]. The qualitative interpretation was as follows: (i) poor if ICC < 0.5; (ii) moderate if 0.5 ≤ ICC < 0.75; (iii) good if 0.75 ≤ ICC < 0.90; and (iv) excellent if ICC > 0.9 [21].

The agreement procedure between the smart trainer and the pedals in each power stage included (i) a comparison of power data and (ii) Bland–Altman plots. Independent samples *t*-test (*p* < 0.05) were used to compare data. Cohen’s d was used to estimate the standardized effect size between devices and was categorized as follows: (i) trivial if 0 ≤ d < 0.20; (ii) small if 0.20 ≤ d < 0.60; (iii) moderate if 0.60 ≤ d < 1.20; (iv) large if 1.20 ≤ d < 2.00; (v) very large if 2.00 ≤ d < 4.00; and (vi) nearly distinct if d ≥ 4.00 [22]. The Bland–Altman analysis included the plots of the difference and the mean values of the two devices [23]. For qualitative assessment, analytical modeling data were considered valid and appropriate if at least 80% of the plots were within the ± 1.96 SD of the difference (95CI). Data analyses were performed using Statistical Package for the Social Sciences v29 (SPSS, IBM, Chicago, IL, USA). Prism GraphPad Prism 8 (Dotmatics, Bishops Stortford, UK) was used for the Bland–Altman analysis.

## 3. Results

Descriptive statistics for both devices per power stage are shown in Table 1. The largest relative difference (poorest accuracy) between the smart trainer and the power target preset in the APP was found at the 100 W stage (0.82 ± 0.95%). The greatest CV verified by the smart trainer was also found at the 100 W stage (0.97%) and the smallest at the 150 W stage (0.20%). As for the pedals, the greatest CV was for the 100 W stage (0.95%), and the smallest was for the 150 W, 210 W, and 270 W (CV = 0.32%). The relative differences between the two devices were small. The largest relative difference (i.e., smallest accuracy) was found at the 100 W (0.31 ± 0.25%) and 130 W (0.31 ± 0.14) stages (Table 1). Regarding reliability, both devices showed strong reliability. Based on the CV, the reliability was considered excellent in all stages. As for the ICC, this ranged between 0.731 in the 150 W stage (moderate) and 0.968 in the 130 W stage (excellent) (Table 1). Cadence tended to increase with the power increase, but the fastest was noted at stage 210 W (smart trainer: 93.3 ± 5.1 rpm; pedals: 93.2 ± 5.2 rpm). Non-significant differences were noted between devices for cadence in all stages.

**Figure 1:**
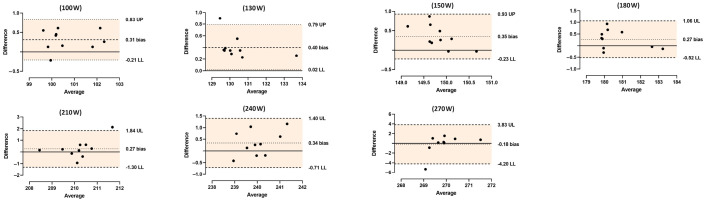
Bland–Altman plots (difference and average) between the TN2T smart trainer and the GV3 pedals for all power stages measured (100 W; 130 W; 150 W; 180 W; 210 W; 240 W; and 270 W). UP—upper limit of the 95% confidence interval; LL—lower limit of the 95% confidence interval.

Independent samples *t*-test comparison showed no significant differences between the devices with trivial (stage 270 W) to moderate (stage 150 W) effect sizes in all power stages (Table 2). Figure 1A–G shows the Bland–Altman plots per power stage. In all of them, the analysis showed that more than 80% of the plots fell within the 95CI intervals. Thus, a strong agreement between devices.

## 4. Discussion

The main objective of this study was to investigate the metrological proprieties of the TN2T (accuracy and reliability), as well as its agreement with the GV3 pedals at different power stages. The main findings indicated that the TN2T presented residual differences to the power target set on the APP and the pedals (i.e., good accuracy). Both devices showed non-significant differences with a strong agreement across the different power stages measured and with an overall excellent reliability.

The literature reports information on the level of validity between power ergometers [17,24]. Overall, the main trend of these results indicates that bicycle power wearables can replace other fixed or laboratory power ergometers with validity. Today, the monitoring of the smart trainers is based on APPs that allow users to increase or decrease their workload wirelessly. As far as we know, there is no information in the literature on whether the power achieved by the smart trainer differs from the power target set on the APP. The results of this study showed a residual relative difference between the defined power target and the power achieved with the smart trainer. This information can be important to understand if the power target set on the APP is the same as the power generated by the smart trainer. It was noted that the largest relative difference between the smart trainer and the preset power target was during the lowest power stage (100 W) and decreased with a power increase. Cyclists tend to have greater cadence variability at their slowest cadence and, consequently, a smaller cadence variability at their fastest cadences [25,26]. This may happen due to several physiological and mechanical factors. It was argued that, during fast cadences, cyclists need to maintain more consistent and efficient neuromuscular coordination to sustain fast cadences. This reduces variability as the athlete settles into a rhythm that maximizes power output and minimizes wasted energy [27]. Indeed, it was shown that cyclists often train at higher cadences to improve their efficiency and endurance [28] and that there is no strong evidence for the benefit of training at low cadences [29]. Therefore, smaller cadence variability at faster cadences can be seen because of improved neuromuscular coordination and adaptations from specific training aimed at efficiently maintaining fast speeds.

As mentioned above, to our knowledge, and as indicated by others [17], there is still no information about the metrological characteristics of the TN2T smart trainer. It was decided to make a comparison between this smart trainer and the GV3 pedals as cyclists (irrespective of the competitive level) can choose to train in both a stationary way and in a field context. The GV3 pedals have been compared to a gold standard Lode Excalibur Sport ergometer [9] and other power meters [24,30], and overall, they are a reliable and accurate measure of power output and cadence, at least during submaximal cycling, as in this study. The descriptive data from our study showed a residual difference between the two devices (relative difference ranging between 0.07% and 0.31%). The agreement was revealed by comparing the data plots of both devices and Bland–Altman analysis. All criteria were met. Curiously, the pedals tended to show smaller (but residual) power values than the smart trainer. Since the point of contact of the force production is at the cyclist’s foot, it could be argued that the highest values would be verified by the pedals, as reported by others [30]. However, based on the residual differences between the two devices, it can be stated that the smart trainer strongly represents the power output obtained by the pedals.

It must be highlighted that the aim of the present study was not to validate the TN2T smart trainer. For such purpose, i.e., validity (which is the ability of a given device to reproduce what it is designed to measure [1,31]), the comparison should be performed with a gold standard device such as the Lode Excalibur ergometer [15] or other cycle-mounted power meter devices that are considered gold standard such as the SRM power meter [8,32]. Once again, the aim of this study was to investigate the metrological proprieties of the TN2T (accuracy and reliability) and its agreement with the GV3 pedals. As aforementioned, the GV pedals showed to be a reliable and accurate measure of power output and cadence during submaximal cycling [24,30]. Notwithstanding, other studies noted somewhat contradictory findings [1,15]. For instance, Lanferdini and co-workers [15] aimed to evaluate and compare the power output reliability, based on two different pedaling protocols (incremental and variable tests), using the GV pedals and a Lode Excalibur ergometer. It was noted that GV pedals did not provide reliable values, which were consistent with those obtained by the Lode Excalibur between 100 and 400 W (in both incremental and variable tests) [15]. The authors indicated that differences in power output measured by the GV pedals did not present a specific pattern and had inconsistent errors. They also argued that a software update could be useful to overcome these errors and minimize the lack of association and agreement between the two studied methods, and it might decrease the coefficient variation, standard error of the measurement, and minimum detectable change values presented by the pedals [15]. Notwithstanding, it must be pointed out that these authors used the first version of the GV pedals (at least based on their description). Similar findings were reported by other authors who used the same first version of the GV pedals [1]. On the other hand, Novak and Dascombe [30] and Nimmerichter and co-workers [24] used the first and the second versions of the GV pedals, respectively, against the SRM power meter. Both studies indicated that the GV pedals did not differ significantly from the SRM power meter and showed good reliability in submaximal efforts. Additionally, Dickinson and Wright [9] used the same version of the GV pedals (GV3) as we did in the present study when comparing their metrological properties against the Lode Excalibur ergometer. These authors reported good reliability outputs like ours for the GV3 pedals. Therefore, one can indicate that the GV3 pedals might be a reliable alternative to the SRM power meters.

The present results indicate that the TN2T smart trainer is an accurate and reliable device for those (i.e., cyclists, coaches, or researchers) who intend to use it in a training or research context for submaximal efforts (at least between 100 W and 270 W). Moreover, based on the residual differences between the two devices within these power stages, it can be assumed that cyclists can use both devices simultaneously without getting misleading results, depending on their preferences, needs, or abilities. Cyclists and researchers can also rely on the smart trainer’s APP. There were only residual differences between the power set on the APP and the power obtained on the TN2T. Once again, it should be mentioned that despite not using a gold standard device for comparison, the GV3 pedals have been shown to be a good alternative to such devices. Therefore, whenever researchers report errors when using the GV pedals, caution should be taken when assuming that this device can compromise the athletes’ training load and consequently their performance.

The main limitations that can be considered are the following: (i) The metrological characteristics refer only to submaximal power stages, i.e., between 100 W and 270 W; (ii) the transition phase between power stages was not considered; and (iii) a small number of participants were recruited. Therefore, future studies could compare the TN2T smart trainer with gold standard ergometers (such as the Lode Excalibur device) or other valid devices (e.g., SRM crankset) to understand its metrological characteristics (including validation, repeatability, and reproducibility). This comparison could be performed with the greatest range of loads or even during sprinting. Transition phases between power stages could also be monitored to understand how much time this smart trainer needs to achieve the preset intensity. Finally, more participants could be included to understand if the results differ from the ones obtained in the present study. Additionally, participants may also include athletes with better performance levels. Notwithstanding, it should be mentioned that the performance level of the participants may not interfere with the device’s outcomes. The main benefit of using better athletes is the opportunity to use such devices at greater loads and cadences.

## 5. Conclusions

Cyclists and researchers can rely on the TN2T smart trainer APP. Based on the study with ten regional-level male cyclists, the results show that there were only residual differences between the power set on the APP and the power obtained on the TN2T. Also, only residual differences between the TN2T smart trainer and the GV3 pedals were noted (i.e., good accuracy). Both devices showed non-significant differences with a strong agreement across the different power stages measured. Therefore, it can be concluded that the TN2T smart trainer is an accurate and reliable device for submaximal power stages (100–270 W).

## Figures and Tables

**Table 1:** Descriptive statistics of the power outputs obtained by the smart trainer and the pedals (mean ± one standard deviation). The coefficient of variation (CV) at each power stage is shown for both devices. It also presents the accuracy (relative difference, in %) of the smart trainer about the target power and between the two devices. The reliability between the two devices for each power stage, measured with the coefficient of variation (CV) and intraclass correlation coefficient (ICC), is also shown.

	TN2T			GV3			Accuracy TN2T vs. APP	Accuracy TN2T vs. GV3	Reliability TN2T vs. GV3	
Power Stage	Mean ± SD	CV (%)	Cadence (rpm)	Mean ± SD	CV (%)	Cadence (rpm)	Relative Difference (%)	Relative Difference (%)	CV (%)	ICC
**100 W**	100.8 ± 1.0	0.97	88.1 ± 7.2	100.5 ± 1.0	0.95	88.1 ± 7.3	0.82 ± 0.95	0.31 ± 0.25	0.15	0.961
**130 W**	130.6 ± 1.2	0.86	86.4 ± 4.0	130.2 ± 1.3	0.92	86.4 ± 4.0	0.45 ± 0.84	0.31 ± 0.14	0.15	0.968
**150 W**	150.0 ± 0.3	0.20	88.1 ± 7.7	149.7 ± 0.5	0.32	88.0 ± 7.5	0.00 ± 0.20	0.24 ± 0.19	0.12	0.731
**180 W**	180.8 ± 1.2	0.62	92.8 ± 5.3	180.5 ± 1.3	0.70	92.8 ± 5.3	0.44 ± 0.62	0.15 ± 0.21	0.08	0.965
**210 W**	210.3 ± 1.1	0.50	93.3 ± 5.1	210.0 ± 0.7	0.32	93.2 ± 5.2	0.14 ± 0.50	0.13 ± 0.56	0.06	0.768
**240 W**	240.2 ± 0.9	0.36	91.2 ± 4.4	239.8 ± 0.7	0.27	91.3 ± 4.5	0.07 ± 0.36	0.14 ± 0.21	0.07	0.842
**270 W**	269.8 ± 1.5	0.53	92.7 ± 2.4	270.0 ± 0.9	0.32	92.7 ± 2.4	0.08 ± 0.54	0.07 ± 0.71	0.03	0.967

**Table 2:** Comparison between the power obtained by the TN2T smart trainer and the GV3 pedals per power stage.

Power Stage	*t*-Test	MD	95CI	*p*	D [Descriptor]
**100 W**	0.68	0.31	−0.65 to 1.27	0.504	0.31 [small]
**130 W**	0.73	0.40	−0.75 to 1.56	0.473	0.33 [small]
**150 W**	1.85	0.35	−0.05 to 0.75	0.081	0.83 [moderate]
**180 W**	0.48	0.27	−0.91 to 1.46	0.634	0.22 [small]
**210 W**	0.66	0.27	−0.60 to 1.14	0.520	0.29 [small]
**240 W**	0.95	0.34	−0.41 to 1.10	0.354	0.42 [small]
**270 W**	−0.31	−0.18	−1.44 to 1.07	0.760	0.15 [trivial]

MD—mean difference; 95CI—95% confidence interval; D—Cohen’s d (effect size indicator).

## Data Availability

Data are unavailable due to privacy or ethical restrictions.
